# Effect of Disease Severity on Respiratory Impedance in Parkinson’s Disease

**DOI:** 10.1177/0972753120960265

**Published:** 2020-11-09

**Authors:** Meghashree Sampath, Achal Kumar Srivastava, Vinay Goyal, Ashok Kumar Jaryal, Kishore Kumar Deepak, Anjana Talwar

**Affiliations:** 1 Department of Physiology, All India Institute of Medical Sciences, New Delhi, India; 2 Department of Neurology, All India Institute of Medical Sciences, New Delhi, India

**Keywords:** Airway resistance, Impulse Oscillometry, motor disorder, Parkinson’s disease, pulmonary function, spirometry

## Abstract

**Background:**

Parkinson’s disease (PD) is a progressive neurodegenerative disorder. PD has been traditionally perceived as a motor disorder. However, it is frequently associated with pulmonary dysfunction which has been assessed by Spirometry, an effort-dependent technique.

**Purpose:**

To evaluate in patients with PD the effect of disease severity on respiratory impedance using Impulse Oscillometry (IOS) and to correlate with Spirometry.

**Methods:**

The study was conducted on 30 patients diagnosed with PD. Pulmonary function was assessed by IOS and spirometer. IOS is an effort-independent technique that uses sound waves of different frequencies to measure airway resistance. Spirometer measures the lung volume and generates flow–volume and volume–time relationship.

**Results:**

The mean age of patients was 60.1±9.45. Resistance at 5 Hz (R5) was found to be negatively correlated with forced expiratory volume in the first second of the FVC manoeuver (FEV_1_) (*r* = –0.628, *P* = .002), FEV_1_/FVC (forced vital capacity) (*r* = –0.487, *P* = .025), and PEF (*r* = –0.599, *P* = .004), and resistance at 20 Hz (R20) with FEV_1_ (*r* = –0.474,

*P* = .029) and PEF (*r* = –0.522, *P* = .015). There was significant increase in R5 (0.32(0.36–0.28) vs 0.47(0.60–0.36); *P* = .04) and R20 (0.25(0.28–0.20) vs 0.30(0.40–0.25); *P* = .04) in stage II as compared to stage I of Hoehn–Yahr scale.

**Conclusion:**

IOS might be a promising tool for diagnosis of respiratory dysfunction in addition to Spirometry, especially in cases where patients are not able to perform forced manoeuvers.

## Introduction

Parkinson’s disease (PD) is a progressive neurodegenerative disorder. It is one of the most important movement disorders. Around 6.3 million people worldwide suffer from PD.^1^ The age of onset is in the 60s (range: 35–85 years), and the course of the illness ranges between 10 and 25 years encompassing both motor and non-motor symptoms.^2^

PD has been traditionally perceived as a motor disorder. However, several non-motor symptoms have gained attention in recent years, including autonomic, sensory, neuropsychiatric and cognitive dysfunction.^3,4^ However, any impairment in pulmonary function has not been generally manifested due to the sedentary lifestyle of the patients, though studies have been reported where pneumologic problems were found to be the most common cause of death,^4^ but these studies have used effort-dependent technique to assess pulmonary function. Though obstructive patterns have been observed by Spirometry and increased resistance by body plethysmography,^5^ it is not clear whether it is due to a motor disability or due to changes in the airway resistance or both.

This study, thus, aims to assess the effect of disease severity on pulmonary function using an effort-independent technique, that is, Impulse Oscillometry (IOS) and correlate with GOLD standard Spirometry to analyze if IOS is better at detecting pulmonary dysfunction in patients with PD.

## Methods

### Study Design

This was a cross-sectional observational study assessing respiratory dysfunction in PD. Consecutive patients visiting the Neurology outpatient department after clinical diagnosis were selected based on inclusion and exclusion criteria from a tertiary care hospital. The study protocol was approved by the Institute Ethics committee (Ref no: RT-3/22.07.2015). Written informed consent was taken from all the subjects.

### Participants

The study was conducted on 30 patients with PD. The disease was diagnosed according to the United Kingdom Brain Bank criteria.^5^ Clinically diagnosed PD patients having Hoehn–Yahr (H-Y) stage from I to IV and age ranging from 40 to 70 years; both male and female were included in the study. Patients with a history of lung or cardiovascular disease affecting pulmonary function and those unable to perform pulmonary function test (PFT) due to anatomical abnormalities were excluded. All patients were non-smokers. Demographic features such as age, sex, height, and weight were noted. Disease characteristics such as time since onset of symptoms and severity (evaluated by H-Y scale) were recorded. Patients were divided into two groups based on the H-Y scale (stage I and stage II) (Table 1).

### Study Procedure

PFT was conducted using Spirometry (Medisoft Spiroair) and IOS System (IOS-Jaeger). Patients were given instructions and demonstrations before the test. Trial sessions were held to get the patients familiarized with the instrument. IOS was performed before Spirometry on the same day. Volume calibration for Spirometry and IOS was done using a 3 L syringe, and pressure calibration for IOS was performed with a reference resistance (0.2 kPa/L/s) daily. After the explanation of the procedure, patients were asked to sit comfortably without legs crossed and with a nose clip. Besides, during IOS maneuvers patients were asked to support their cheeks with hands to prevent shunting of impulses, followed by normal tidal breathing in a relaxed state for at least 30–45 s during which around 120–150 sound impulses/pressure oscillations were pushed into the lungs from which different parameters were calculated.

**Table 1.** Demographic Data of Patients

	Patients		
	Pooled Patients *N* = 30	Stage I *N* = 7	Stage II *N* = 7
Severity of PD	H-Y Stage I, II, III, IV	H-Y Stage I	H-Y Stage II
Age	60.1±9.45	55.2±8.15	59.71±9.89
Height (cm)	164.5±6.80	166±3.91	163±5.85
Weight (kg)	64.34±12.29	67±14.3	60.57±11.70
BMI	23.5±3.93	24.40±5.39	22.48±3.73

**Source:** The authors.

**Note:** Data has been represented as mean±SD.

**IOS System** is a non-invasive and effort-independent technique, unlike Spirometry which requires active participation by the patient.^6^ Dubois et al., in 1956 first described the forced oscillation technique (FOT) as a tool to measure respiratory impedance using the application of external pressure waves of single or multiple frequencies (2–35 Hz) to the spontaneous tidal breathing of patient.^6,7^ IOS is a form of FOT which uses pressure oscillations at a fixed frequency (square wave).^6^ Inspiratory and expiratory pressure and flow measured by the respective transducers are separated from breathing pattern by “signal filtering.”^8^ Respiratory impedance (Zrs) includes both respiratory resistance (Rrs) and respiratory reactance (Xrs) which are calculated by Fast Fourier Transformation (FFT) over a range of frequencies.^7^

Higher frequencies (>20 Hz) travel deeper into the lung and distal airways, whereas lower frequencies (<15 Hz) reflect from proximal airways. Resistance and reactance at 5 Hz and 20 Hz are denoted as R5, R20, and X5, X20 respectively. Therefore, resistance at lower frequency, that is, 5 Hz (R5) gives information about the total respiratory system, resistance at higher frequency, that is, 20 Hz (R20) provides information about central airways, and the difference between R5 and R20 reflects peripheral/small airways.^8^ Either central or peripheral airway obstruction results in increased R5. Central obstruction elevates resistance evenly; therefore, it is independent of frequency. Peripheral obstruction elevates resistance at a lower frequency; therefore, resistance is frequency dependent.^8^ Resistance is the in-phase component of respiratory impedance that reflects forward pressure of conducting airways, whereas reactance is the out-of-phase component reflecting capacitive and inertive properties of airways.^6^ Capacitance represents the elastic properties of the lung and inertance represents the mass inertia of the moving air column. Reactance can be thought of rebound resistance giving information about the small airways. Conventionally, capacitance is denoted by negative value and inertance by a positive value. At lower frequency, capacitive pressure loss dominates; therefore, reactance at 5 Hz (X5) gives information about tissue elastance and distal airways, whereas at higher frequency, inertive pressure loss dominates. As the elasticity of the lung decreases, capacitance becomes more negative. The frequency at which the total reactance is zero, that is, the magnitude of capacitance and inertance are the same, is known as resonant frequency (Fres).^6^ Area of reactance (Ax) represents total reactance at all frequencies between 5Hz and Fres and provides information about the distal/peripheral airways. Normal value of Ax is <0.33 kPa/l.^6,8^ Coherence has a value between 0 and 1 reflecting reproducibility of measurements. For accurate testing, at 5 Hz coherence should be >0.8 cm H_2_O and at 20 Hz, it should be between 0.9 and 1.^8^ Average of 3–4 technically acceptable recordings were considered for calculations.

**Spirometry** maneuver was performed according to the guidelines given by Miller et al.^9^ Best of three technically accepted tests were obtained and forced vital capacity (FVC), forced expiratory volume in the first second of the FVC manoeuver (FEV_1_), a ratio of FEV_1_ to FVC (FEV_1_ % FVC), and peak expiratory flow (PEF) were calculated. All lung volumes were expressed as a percentage of predicted values.

### Statistical Analysis

Each parameter was tested for distribution of the data based on standard normality tests (D’ Agostino–Pearson omnibus normality test and Shapiro–Wilk test). The independent variable was stage I and stage II. Dependent variables were parameters of IOS and parameters of Spirometry. Two group comparisons were done using unpaired t-test and Mann–Whitney U test, as appropriate. To study the relationship between IOS and Spirometry techniques, Spearman’s correlation was used. The level of statistical significance was set at *P* < .05. All statistical analyses were performed using GraphPad Prism version 5.00 for Windows (GraphPad Software, Inc., USA).

## Results

A total of 30 patients participated in the study (Table 1). All patients were on medication during the recording. Spirometry maneuver could not be performed in 9 out of 30 patients due to a high rate of tremors; so, data for remaining patients have been presented. R5 was found to be negatively correlated with FEV_1_ (*P* = .002), FEV_1_/FVC (*P *= .025), and PEF (*P* = .004) and R20 with FEV_1_ (*P* = .029) and PEF (*P* = .015) (Table 2).

**Table 2.** Correlation of Spirometry and IOS Parameters in PD Patients (*n* = 21)

	R5 (kPa/l/s)	R20 (kPa/l/s)
FEV_1_% pred	–0.628**	–0.474*
FEV_1_/FVC	–0.487*	–
PEF% pred	–0.599**	–0.522*

**Source:** The authors.

**Note:** Level of significance is denoted by asterisk (*) and (**) at 5% and 1% respectively.

Data was stratified based on disease severity. There was a significant increase in R5 (*P* = 0.04) and R20 (*P* = 0.04) in stage II as compared to stage I. However, we could not observe any significant differences in Spirometric parameters (Table 3).

**Table 3.** Parameters of IOS and Spirometry in Stage I and Stage II

	Stage I (*n* = 7)	Stage II (*n* = 7)	*P* Value
R5 (kPa/l/s)	0.32(0.36-0.28)	0.47(0.60-0.36)	0.04*
X5 (kPa/l/s)	–0.13(–0.10) – (–0.16)	–0.13(–0.11) – (–0.29)	0.94
R20 (kPa/l/s)	0.25(0.28-0.20)	0.30(0.40-0.25)	0.04*
Ax (kPa/l)	0.67(0.84-0.22)	0.66(2.50-0.41)	0.70
Fres (Hz)	17.27 (19.49-16.10)	17.41(24.04-16.39)	0.43
Z5 (kPa/l/s)	0.35(0.38-0.32)	0.49(0.66-0.38)	0.07
FEV_1_% pred	95(100.5-76.30)	76.8(88.7-38.7)	0.10
FVC% pred	99.9(107.9-78.5)	80.8(98.6-59.8)	0.26
FEV_1_/FVC	76.08 (76.97-74.83)	70.99(78.79-45.09)	0.38
PEF% pred	55.20 (85.40-37.60)	41.20967.40-16.20)	0.34

**Source:** The authors.

**Note:** Data has been represented as median (inter quartile range). Level of significance is denoted by asterisk (*) at 5%.

## Discussion

Despite recognition of pulmonary involvement in PD quite early, little is known about the existing respiratory dysfunction. This study elucidates the respiratory abnormalities in PD using IOS and Spirometer. There was not much difference in the mean percentage predicted values of Spirometric parameters in those who were able to perform the tests, except for a consistent dip in the PEF among all patients. On analyzing individual records, we observed three restrictive, three obstructive, and four mixed patterns.

Lower FEV_1_, FEV_1_/FVC ratio, and PEF has been a frequent observation in PD patients.^10,11,12^ The reduction in the force exerted by expiratory muscles is discernible in the huge dip in the PEF.^13^ The underlying motor disability, eventually results in low chest wall compliance and increased chest wall rigidity^13^ leading to disordered respiratory mechanics, significantly contributing to increased morbidity and mortality in PD.^14^

The negative correlation of total and proximal airway resistance R5 and R20, respectively, with FVC, FEV_1_, and PEF has been established in few studies. Qi et al.^15^ suggested that R5 might be used as a tool to investigate airway obstruction in asthmatics, whereas Kolsum et al.^16^ found reactance values to be more significantly correlated to Spirometric parameters in COPD patients.

The total airway resistance, that is, R5 was found to be elevated with increasing severity of the (higher H-Y stage versus lower H-Y stage) disease. To the best of our knowledge there is no study to corroborate the current findings as this is the first study to assess pulmonary function with increasing disease severity using the effort independent technique. However, Spirometric parameters could not pick up any significant difference with increasing disease severity.

We performed both the techniques in our study, one requiring effort (Spirometer) and the other which is independent of effort (IOS) to rule out the muscular component which is the underlying disability of patients with PD. We have found that in spite of the decreased muscular force, these patients have high resistance as measured by IOS. So, the pulmonary dysfunction reported in these patients might not be completely because of motor disability.

## Conclusion

Resistance at 5 Hz and 20 Hz might be used as a diagnostic tool in conjunction with Spirometry in assessing the pulmonary dysfunction with increasing severity of the disease and also in cases where patients are not able to perform forced maneuvers.
